# Quantitative analysis of amino acid excretion and consumption by *Methanothermobacter marburgensis* in fed-batch cultivation mode

**DOI:** 10.1007/s00726-026-03498-1

**Published:** 2026-02-02

**Authors:** Barbara Reischl, Benjamin Schupp, Christian Fink, Simon K.-M. R. Rittmann

**Affiliations:** 1 Archaea Physiology & Biotechnology Group, Department of Functional and Evolutionary Ecology, Universität Wien, Djerassiplatz 1, 1030 Wien, Austria; 2 ACIB – Austrian Centre of Industrial Biotechnology, Wien, Austria; 3 Arkeon GmbH, Tulln an der Donau, Austria

**Keywords:** Microbiology, Biotechnology, Anaerobes, Bioreactor, Bioprocess, Gas fermentation, Archaea, Methanogens

## Abstract

The methanogenic archaeon *Methanothermobacter marburgensis* offers a promising alternative to traditional bacterial systems for the sustainable production of proteinogenic amino acids (AAs), eliminating the need for sugar-based feedstock. In this study, we quantitatively examined AA excretion and consumption in fed-batch cultivation mode in bioreactors under varying ammonium (NH_4_^+^) concentrations and gas compositions. *M. marburgensis* demonstrated excretion of a wide spectrum of AAs with distinct profiles shaped by nitrogen availability. While high NH_4_^+^ concentrations suppressed total AA excretion, NH_4_^+^ limited conditions triggered alanine accumulation followed by its re-assimilation, suggesting a regulatory mechanism linked to nitrogen stress. Moreover, carbon limitation and nitrogen excess resulted in the production of an AA pattern including asparagine. Despite lower overall productivity compared to engineered bacterial strains, *M. marburgensis* exhibited the unique ability to simultaneously excrete multiple AAs without requiring organic carbon input. These findings advance the feasibility of using methanogens for AA bioprocessing and the development of archaea as next-generation microbial cell factories.

**Supplementary Information:**

The online version contains supplementary material available at 10.1007/s00726-026-03498-1.

## Introduction

The biotechnological production of proteinogenic amino acids (AAs) through heterotrophic microbial fermentation has already been practiced for decades (Kinoshita 1972). Today, this field represents a multibillion-dollar global industry and an established pillar of biotechnology (Wendisch 2020). Industrial AA production is a growing market, as the worldwide demand increases annually by about 8.3% from 2024 to 2034, which is driven by e.g., applications in food, feed and materials industries (Bidwai 2024; Wendisch 2020). The well-studied model organisms for AA production are *Corynebacterium glutamicum* and *Escherichia coli*. They have been genetically engineered to serve as microbial cell factories for optimized large-scale production of single AAs (Becker et al. 2011; Becker and Wittmann 2012; D’Este et al. 2018; Ding et al. 2023; Liu et al. 2022; Lei Wang et al. 2024a, b; Lijuan Wang et al. 2024a, b). However, besides further development of existing cell factories, the interest has shifted towards production of specialized AAs or AA mixtures (Lee and Wendisch 2017; Wendisch 2020; Wolf et al. 2021).

Methanogenic archaea (methanogens), such as *Methanothermobacter marburgensis*, *Methanocaldococcus villosus* and *Methanothermococcus okinawensis*, are able to synthesize and excrete AAs into the growth medium simultaneously and, by applying different process parameters for their cultivation, different AA patterns may be obtained (Reischl et al. 2025; Rittmann et al. 2023a, b, c; Taubner et al. 2023, 2019). Furthermore, methanogens present an important advantage for sustainable AA production compared to *E. coli* and *C. glutamicum*, as they do not require sugar-based feedstock. In general, methanogens are specifically known for their importance in the anaerobic part of the global carbon cycle, especially for the ability to generate methane (CH_4_) as the end product of their energy metabolism by e.g., reduction of carbon dioxide (CO_2_) with molecular hydrogen (H_2_) (Lyu et al. 2018). Furthermore, certain methanogens harbor the ability to fix molecular nitrogen (N_2_) (Belay et al. 1984; Leigh 2000; Maslać et al. 2022; Murray and Zinder 1984; Reischl et al. 2025).

In this study, we have chosen the methanogenic archaeon *M. marburgensis*, due to its biotechnological relevance. *M. marburgensis* harbors a high specific growth rate paired with high CO_2_-fixation rates and high-performance CH_4_ production capabilities (Abdel Azim et al. 2017; Mauerhofer et al. 2021; Rittmann et al. 2018; Seifert et al. 2014). Moreover, various set-ups have already been used for cultivation and bioprocess development of *M. marburgensis* in closed batch, fed-batch and continuous culture mode (Abdel Azim et al. 2017; Hofmann et al. 2025; Seifert et al. 2014). Additionally, there are tools available for genetic modification of *M. marburgensis* (Klein et al. 2025), it has been proven that *M. marburgensis* is capable of excreting AAs (Taubner et al. 2023), it is able to fix N_2_ for AA secretion (Reischl et al. 2025) and can be grown at high pressure conditions (Mauerhofer et al. 2021; Orthofer et al. 2025; Pappenreiter et al. 2019). In contrast to previous reports that primarily examined AA secretion by methanogens in closed batch cultivation mode (Reischl et al. 2025; Taubner et al. 2023), this study investigates AA excretion and re-uptake of AAs by *M. marburgensis* in fed-batch cultivation mode. By systematically varying the partial pressures of H_2_/CO_2_/N_2_ under different availabilities of ammonium (NH_4_^+^), we aimed to examine how gas composition and nitrogen supply affect the AA excretion patterns and rates as well as the re-uptake of AAs.

## Materials and methods

### Strains

*Methanothermobacter marburgensis* DSM 2133 (Schönheit et al. 1979; Wasserfallen et al. 2000) was used in all experiments.

### Chemicals

H_2_ (99.999 Vol.-%), CO_2_ (99.999 Vol.-%), N_2_ (99.999 Vol.-%), H_2_/CO_2_ (20 Vol.-% CO_2_ in H_2_) (4:1), H_2_/CO_2_/N_2_ (11.13 Vol.-% N_2_ and 11.13 Vol.-% CO_2_ in H_2_) (7:1:1) were used for pre-cultures and for fed-batch experiments. For gas chromatography (GC), N_2_/CO_2_ (20 Vol.-% CO_2_ in N_2_), CH_4_ (99.995 Vol.-%) and the standard test gas (Messer GmbH, Wien, Austria) (containing 0.01 Vol.-% CH_4_, 0.08 Vol.-% CO_2_ in N_2_) were additionally used. All gases, except the standard test gas, were purchased from Air Liquide (Air Liquide GmbH, Schwechat, Austria). All other chemicals were of highest grade available.

### Media

Fed-batch cultivations with *M. marburgensis* were performed on minimal medium (Schönheit et al. 1979), adjusted according to cultivation-specific requirements (Abdel Azim et al. 2017; Rittmann et al. 2012). For inoculation of bioreactors, a stock culture that has been adapted to fed-batch cultivation was used. Fed-batch cultivations were performed at different NH_4_^+^ concentrations (0%, 1%, 5%, 10% and 100%) (Supplementary Table 1) in relation to original media composition of 2.1 g L^− 1^ (Rittmann et al. 2012). Experiments were performed with Na_2_CO_3_ medium (Rittmann et al. 2012) and in a medium without Na_2_CO_3_ (carbonate-free medium) where Na_2_CO_3_ in carbonate-free media was replaced by equal molarities of NaCl (Abdel Azim et al. 2017).

### Fed-batch experiments

All fed-batch experiments were performed with *M. marburgensis* in triplicates (*n* = 3) with H_2_/CO_2_/N_2_ (7:1:1) in DASGIP^®^ 2.2 L bioreactor system (SR1500ODLS, Eppendorf AG, Hamburg, Germany) with 1.5 L working volume of medium including 100 µL L^− 1^ of antifoam (Struktol SB2023, Schill und Seilacher, Hamburg, Germany). Best growth conditions are a temperature of 65 °C and pH of 7 (Abdel Azim et al. 2017; Rittmann et al. 2018). Fed-batches with and without Na_2_CO_3_ in the medium and fed-batches performed with H_2_/CO_2_ (4:1) served as reference. Gassing of N_2_ and CO_2_ was controlled via the MX4/4 unit (Eppendorf AG, Hamburg, Germany). H_2_ gas flow was controlled via the C100L Unit (Sierra Instruments, Monterey, USA). Redox potentials and pH values were monitored by individual Redox- and pH-probes (Mettler Toledo GmbH, Wien, Austria). Before inoculation, the bioreactor was gassed with the respective gas (H_2_/N_2_/CO_2_ or H_2_/CO_2_) to ensure that the conditions inside the bioreactor are anaerobic. After anaerobization 5 mL of 0.5 mol L^− 1^ Na_2_S·9H_2_O were added and the bioreactor was inoculated with 30 mL *M. marburgensis* suspension to the respective starting optical density (OD) (λ = 578 nm, blanked with Milli-Q water) (Beckman Coulter, DU 800 spectrophotometer, California, USA). Then immediately 0.5 mol L^− 1^ Na_2_S·9H_2_O feeding of 0.2 mL h^− 1^ was started and the agitation speed was set to 1600 rpm. Gas and liquid samples were taken after approximately 0, 13, 16, 19, 22 and 25 h of cultivation. Growth was measured spectrophotometrically via OD. Liquid samples of 1 mL for AA analysis were taken at each time point and centrifuged at 16,100 rcf for 30 min at room temperature (5415 R, Eppendorf AG, Hamburg, Germany). Cell pellets and supernatant of each experiment were stored separately in sterile Eppendorf tubes at -20 °C until further analysis.

### Ammonium determination

NH_4_^+^ concentrations were spectrophotometrically quantified using the indophenol blue method, as reported before (Reischl et al. 2025). Calibration was performed with standard ammonium chloride solutions in the same concentration range as the samples.

### Amino acid analysis

For AA analyses, the supernatant of liquid samples was diluted with Milli-Q water at a ratio of 1:4 (to be in the range of the standards), as reported before (Taubner et al. 2019). All AA measurements were performed by HPLC after pre-column derivatization with o-phthalaldehyde (OPA). Detection of AAs was performed fluorometrically. GABA served as internal standard.

### Gas chromatography and analysis

Off-gas sampling was done as previously described (Reischl et al. 2018). Gas samples were taken after approximately 0, 13, 16, 19, 22 and 25 h. The off-gas composition (H_2_, CO_2_, CH_4_ and N_2_) of the collected gas samples was analyzed by using the Agilent Gas Chromatograph (Agilent 7890 A GC, Agilent Technologies, Santa Clara, CA, USA) with a thermal conductivity detector and a 19,808 Shin Carbon ST Micropacked Column (Restek GmbH, Bad Homburg, Germany) as described before (Abdel Azim et al. 2017; Taubner and Rittmann 2016). All measurements were performed at room temperature.

### Statistical analysis

Statistical analyses were performed in R (v4.3.2) using the lawstat and stats packages (Hui et al. 2008). Variance homogeneity was assessed using the Brown-Forsythe test. A two-way ANOVA was applied to analyze the effects of cultivation time, NH_4_^+^ concentration and the interaction of time and NH_4_^+^ concentration of alanine (Ala), asparagine (Asn), gluamic acid (Glu) and glycine (Gly). A significance level of α = 0.05 has been applied for the Brown-Forsythe tests and for the two-way ANOVAs.

## Results

### Growth of *M. marburgensis* in fed-batch cultivation mode

**Fig. 1:**
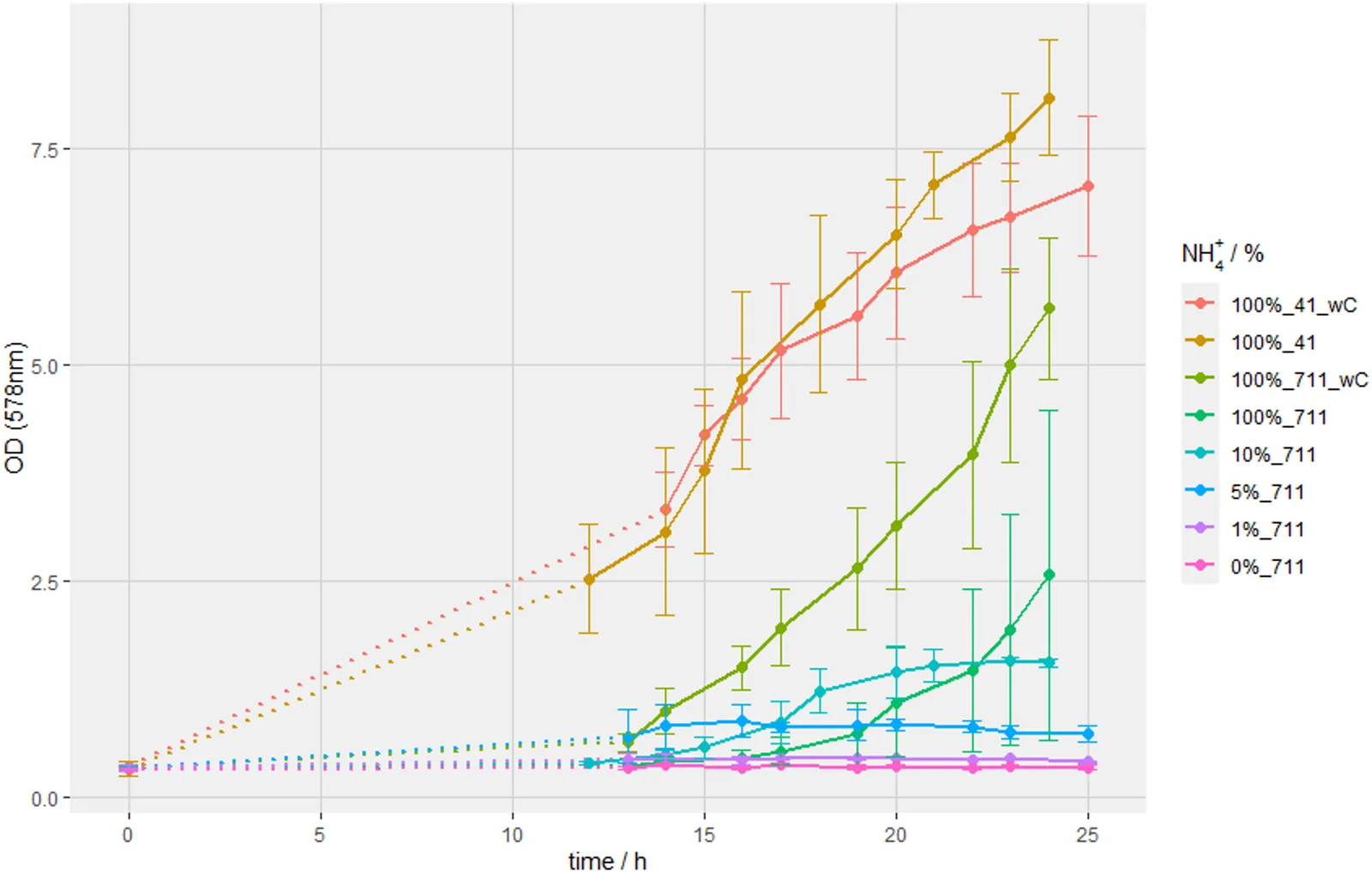
Growth kinetics of *M. marburgensis* with varying NH_4_^+^ concentrations in different media with Na_2_CO_3_ (wC) and without Na_2_CO_3_ gassed with either H_2_/CO_2_ (4:1) (41) or with H_2_/CO_2_/N_2_ (7:1:1). Gassing with H_2_/CO_2_ (4:1) as well as the addition of Na_2_CO_3_ in the media served as reference growth experiments. Dashed lines represent unsupervised overnight growth. The legend on the right-hand side indicates the percentage of the NH_4_^+^ concentration from 0%, 1%, 5%, 10% and 100%. The numbers 41 or 711 refer to the ingas flow compositions of H_2_/CO_2_ (4:1) and H_2_/CO_2_/N_2_ (7:1:1), respectively

Fed-batch experiments at NH_4_^+^ concentrations of 0%, 1%, 5%, 10% and 100% (*n* = 3) were performed with *M. marburgensis* in bioreactors (Fig. 1). Growth of *M. marburgensis* on H_2_/CO_2_ (4:1) with carbonate (Na_2_CO_3_) and without Na_2_CO_3_ in the medium resulted in growth up to an OD_578_ of 7.0 and 8.1, respectively, which served as reference runs to earlier findings (Abdel Azim et al. 2017). The experiments with *M. marburgensis* grown on H_2_/CO_2_/N_2_ (7:1:1) served as the actual experiments for assessment of AA excretion. The experiments with H_2_/CO_2_/N_2_ (7:1:1) and 100% NH_4_^+^ with Na_2_CO_3_ in the medium resulted in a 2.2-fold higher OD_578_, compared to results in the medium without Na_2_CO_3_. When we reduced the NH_4_^+^ concentration to 10%, we observed a final OD_578_ of 1.6 after 20 h of incubation. With a reduction to only 5% of the original NH_4_^+^, stationary growth phase with a final OD_578_ of 0.9 was already reached after 15 h of incubation. Experiments with 0% or 1% NH_4_^+^ concentration did not yield in detectable growth of *M. marburgensis* (Fig. 1).

### Amino acid excretion and consumption by *M. marburgensis*

**Fig. 2:**
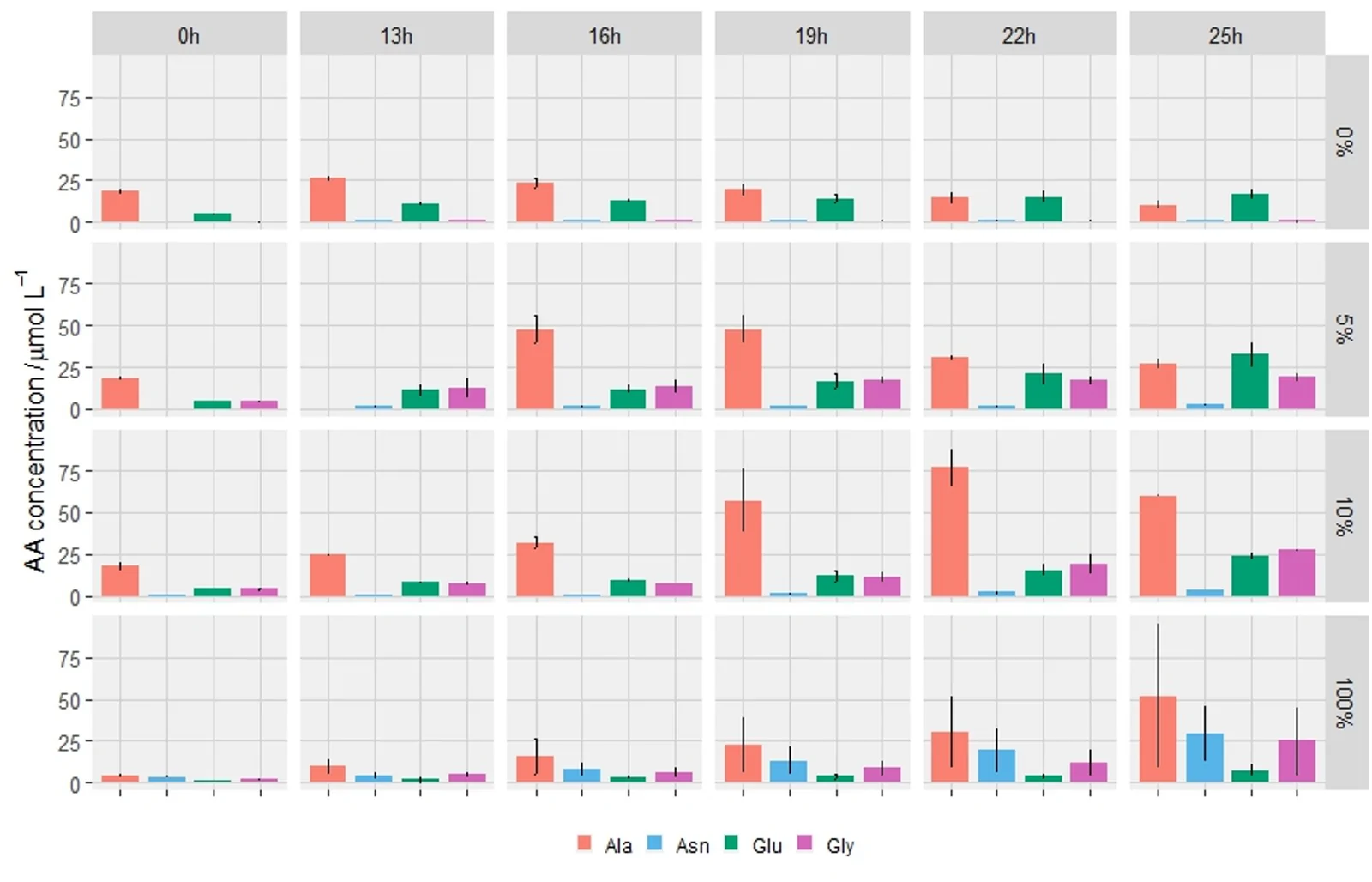
Proteinogenic amino acid (AA) concentration / µmol L^− 1^ of Ala, Asn, Glu and Gly supernatant samples from *M. marburgensis* fed-batch cultivations with H_2_/CO_2_/N_2_ (7:1:1) without Na_2_CO_3_. The AA concentrations are shown as individual bar charts with standard deviations for each time point (*n* = 3). The legend at the bottom of the graph indicates the quantified AAs. On the left-hand y-axis, the AA concentration / µmol L^− 1^ is shown. Initial NH_4_^+^ concentrations of the respective time series are indicated on the right-hand y-axis from top to bottom: 0%, 5%, 10% and 100%. The sampling time is shown on the x-axis as headers from left to right

We performed a quantitative analysis of AA excretion and re-uptake thereof by *M. marburgensis* from fed-batch cultivations with 0%, 5%, 10% and 100% NH_4_^+^ content and the gas mixture of H_2_/CO_2_/N_2_ (7:1:1) without Na_2_CO_3_ supplementation (Fig. 2). Results of all quantified AAs can be found in Supplementary Fig. 1.

We identified all AAs, except for cysteine, in the analyzed samples excreted by *M. marburgensis*. The concentration of individual AAs varied throughout the experiments and within the different conditions dependent on the growth phase and residual NH_4_^+^ concentration (Fig. 2, Supplementary Fig. 1). We found that Ala, Asn, Glu and Gly were excreted in highest quantities among all AAs (Fig. 2). ANOVA analyses confirmed that the cultivation time had a highly significant effect on the concentration of Ala (Supplementary Table 2), Asn (Supplementary Table 3), Glu (Supplementary Table 4) and Gly (Supplementary Table 5). Furthermore, ANOVA indicated that the NH_4_^+^ concentration had a highly significant effect on the concentration of Ala (Supplementary Table 2), Asn (Supplementary Table 3) and Glu (Supplementary Table 4). Moreover, ANOVA showed that the interaction of cultivation time and the NH_4_^+^ concentration had a very significant effect on the concentration of Asn (Supplementary Table 3) and a highly significant effect on the concentration of Glu (Supplementary Table 4). The highest Ala and Glu concentrations were observed in a state of low NH_4_^+^ levels at the transition to stationary growth phase with a peak volumetric productivity of 2.67 µmol L^− 1^ h^− 1^ for Ala at 5% NH_4_^+^. This state is best visible at 19 h and 22 h of cultivation at 5% and 10% of NH_4_^+^, respectively (Fig. 2). After a complete NH_4_^+^ consumption to values below the detection limit (Supplementary Fig. 2), we observed a reoccurring Ala uptake in all 5% and 10% fed-batch cultivations (Fig. 2, Supplementary Fig. 1). The 0% NH_4_^+^ concentration fed-batch cultivation served as a control to define the background AA concentrations. While we found Ala consumption in the 5% and 10% NH_4_^+^ experiments, the Glu and Gly concentrations continuously increased throughout the fed-batch cultivations.

Compared to the 5% and 10% NH_4_^+^ fed-batch cultivations, *M. marburgensis* excreted a different AA pattern at 100% NH_4_^+^. We observed delayed AA excretion accompanied by lower volumetric productivity compared to 5% and 10% NH_4_^+^ (Fig. 2, Supplementary Fig. 1). Additionally, the Ala, and especially the Glu concentrations were lower compared to the peak concentrations in 5% and 10% NH_4_^+^. However, the Asn concentrations clearly increased throughout the experiment towards far exceeding levels compared to all other NH_4_^+^ concentrations leading to a representative AA pattern change resulting in a shift in AA excretion from Ala and Glu to Asn. Moreover, compared to 5% and 10% NH_4_^+^, 100% NH_4_^+^ in the medium showed less AA production in terms of total excreted AAs (Supplementary Fig. 3) at higher available concentrations of NH_4_^+^ (Supplementary Fig. 2) and at a higher NH_4_^+^ uptake rate (Supplementary Fig. 4). In the experiments with 0% and 5% NH_4_^+^ a decrease in total AA concentration is indicated directly at the onset of the experiment, whereas in the experiment with 10% NH_4_^+^ a higher total amount of AA compared to the 100% NH_4_^+^ experiment was visible (Supplementary Fig. 3).

## Discussion

In this study, we aimed for characterization of AA excretion at differing H_2_ and CO_2_ partial pressures combined with different initial NH_4_^+^ concentrations. We found that the CO_2_ supply is the limiting factor for growth of *M. marburgensis* when the H_2_/CO_2_ ratio is changed from 4:1 (H_2_/CO_2_) to 7:1:1 (H_2_/CO_2_/N_2_). The limitation was proven through the addition of Na_2_CO_3_ supplementation which restored wild-type-like growth (Fig. 1). Additionally, we observed that a sub-optimal supply of CO_2_ combined with NH_4_^+^ limitation had favorable effects on Ala and Glu secretion until the stationary growth phase was reached. Sub-optimal supply of CO_2_ without NH_4_^+^ limitation, however, led to an excretion of Asn and therefore a shift of the AA pattern from Ala and Glu to Asn (Fig. 2). In addition, we confirmed the previously reported Ala uptake by *M. marburgensis* at 5% and 10% of NH_4_ ^+^ of earlier closed batch cultivations under N_2_-fixing conditions (Reischl et al. 2025), which occurred also in the respective fed-batch cultivations at NH_4_ ^+^ concentrations of 5% and 10% in this study. Thus, Ala might be used as a putative carbon and nitrogen source by *M. marburgensis* in the early stationary growth phase under NH_4_^+^-limited conditions when N_2_ is available. It might be possible that in *M. marburgensis* the secretion and the re-uptake of Ala allows for N_2_-fixation in the presence of NH_4_^+^. This effect is known from *Methanococcus maripaludis*, which is capable of N_2_-fixation in the presence of NH_4_ ^+^ and Ala, but not solely on NH_4_ ^+^ without Ala (Lie and Leigh 2002). However, despite the presence of N_2_ and AAs in the growth medium, no clearly detectable increase in OD of *M. marburgensis* was observed at NH_4_ ^+^ concentrations of 0% and 1% (Fig. 1). However, a change in the AA concentrations could be observed at a NH_4_ ^+^ concentration of 0% (Fig. 2). The change of AA excretion by reducing the partial pressure of CO_2_ combined with different concentrations of NH_4_^+^ in fed-batch cultivations is thus a novelty.

In previous studies with *M. marburgensis* and other methanogens, such as *Methanocaldococcus villosus* or *Methanothermococcus okinawensis*, it has been shown that different patterns of AAs can be excreted (Taubner et al. 2019, 2023). Additionally, it was demonstrated that in closed batch cultivations with *M. marburgensis* under N_2_-fixing conditions the volumetric AA productivity and the defined patterns of excreted AAs can be altered (Reischl et al. 2025). The statistically significant increases in Ala and Glu under carbon and nitrogen starvation conditions are also backed by the findings from earlier closed batch cultivations (Reischl et al. 2025). Here, we proved that this finding is also scalable to 1.5 L fed-batch cultivation mode in bioreactors. Especially, the Ala excretion sparked our interest since it has been postulated before that *M. marburgensis* could be able to use Ala as a nitrogen and potentially carbon source in addition to NH_4_^+^ and CO_2_ (Reischl et al. 2025). In this study, we showed that there is a clear re-uptake of Ala under nitrogen starvation (Fig. 2, Supplementary Fig. 1, Supplementary Fig. 2). This finding is also in line with earlier findings from other methanogens, such as *Methanococcus maripaludis*, known to use Ala as an alternative nitrogen source in addition to NH_4_^+^ (Lie and Leigh 2002; Moore and Leigh 2005; Thevasundaram et al. 2022).

A special case marks the condition where NH_4_^+^ is applied in excess while CO_2_ is still supplied at sub-optimal levels. Here, we observed a clear shift of the AA pattern from Ala and Glu to Asn. Asn is synthesized from pyruvate over Asp together with glutamine (Gln) from Glu (Reitzer 2004). Our hypothesis for excess Asn synthesis and excretion with reduction of Ala and Glu is, that pyruvate for Ala biosynthesis is used for asparagine (Asp) production and Glu reacts via Gln to Asn together with Asp. *M. marburgensis* is a prototrophic organism and thus capable of synthesizing all AAs. Thus, the enzymatic machinery for AA inter-conversion is in principle available, as shown in this study and in an earlier study (Reischl et al. 2025). In this way, the shift of AA patterns is explainable, but further transcriptomic or proteomic studies need to follow to confirm our hypothesis. In plants, Asn is known to be a nitrogen storage compound under carbon limitations as Asn has a high carbon to nitrogen ratio among the AAs (Gaufichon et al. 2010). In bacteria and archaea there is only limited knowledge about the function of Asn during carbon limited conditions. It is known, however, that the Asn biosynthesis is highly regulated through allosteric inhibition in *E. coli* (Zampieri et al. 2019).

Various sectors from food industry to packaging are using AA for their products and the worldwide demand for AA, and especially the demand for the animal feed market, is expected to grow over the next years (Wendisch 2020). Therefore, the production of AA from other sources than sugar, for example from CO_2_ by using methanogens has created attraction in the last years (Pfeifer et al. 2021; Reischl et al. 2025; Rittmann et al. 2023a, b, c; Taubner et al. 2023, 2019). For industrial purposes, AA production by *M. marburgensis* is far from being competitive regarding the results presented in this study compared to the genetically engineered bacteria *C. glutamicum* or *E. coli*. In addition, these bacteria are employed to mainly produce single AAs (Becker et al. 2011; Becker and Wittmann 2012; Ding et al. 2023; Liu et al. 2022; Wang et al. 2024a, b; Wolf et al. 2021). Optimized *C. glutamicum* or *E. coli* typically produce AAs at volumetric production rates between 10 and 100 mmol L^− 1^ h^− 1^ (Becker et al. 2011; Ding et al. 2023; Liu et al. 2022). In this study, AA productivities in the range of 1 to 3 µmol L^− 1^ h^− 1^ have been obtained with wild-type *M. marburgensis*. However, a genetically-engineered *M. marburgensis* strain was already shown to be capable of producing leucine at a volumetric productivity of 0.5 mmol L ^− 1^ h ^− 1^ (Fink et al. 2025).

As seen here, and in earlier studies (Reischl et al. 2025; Taubner et al. 2023), *M. marburgensis* is capable to generate and excrete almost all 20 AAs simultaneously. By shaping the AA profile through temperature, substrate concentration or physiological properties (Reischl et al. 2025; Taubner et al. 2023), the production of different AA patterns by methanogens might become of interest to the industrial AA production sector. For instance, the Umami taste is mostly attributed to Glu, Ala and Asp (Zhao et al. 2016). Also, the AA demand for media of cultivated meat consists of certain AA profiles, which leads to more stability, better growth or other properties of cell culture and media (Ardö 2006; O’Neill et al. 2021).

In conclusion, our study shows the capability of *M. marburgensis* to produce different AA patterns based on the NH_4_^+^ concentration in fed-batch cultivation mode in bioreactors. AA production by *M. marburgensis* can now be improved by combined efforts of systems and synthetic biotechnology, bioprocess optimization and scale-up in bioreactors.

## Supplementary Information

Below is the link to the electronic supplementary material.

Supplementary Material 1

## Data Availability

All data are available at the repository of the University of Vienna: https://phaidra.univie.ac.at/o:2140855.
