# Supplementary material for: Quantitative analysis of amino acid excretion and consumption by Methanothermobacter marburgensis in fed-batch cultivation mode
Source: Amino Acids. 2026 Feb 2;58:9. doi: 10.1007/s00726-026-03498-1 (PMC12881071; doi:10.1007/s00726-026-03498-1)
Supplement: Supplementary file 1 [file 726_2026_3498_MOESM1_ESM.pdf]

Supplementary material to: Quantitative analysis of amino acid excretion and consumption by *Methanothermobacter marburgensis* in fed-batch cultivation mode

Barbara Reischl[1,2], Benjamin Schupp[1], Christian Fink[2,3], Simon K.-M. R. Rittmann[1,2,*]

[1]Archaea Physiology & Biotechnology Group, Department of Functional and Evolutionary Ecology, Universität Wien, Wien, Austria

[2]ACIB – Austrian Centre of Industrial Biotechnology, Wien, Austria

[3]Arkeon GmbH, Tulln a.d. Donau, Austria

*Corresponding author:
Dr. Simon K.-M. R. Rittmann, Privatdoz.
Archaea Physiology & Biotechnology Group
Department of Functional and Evolutionary Ecology
Universität Wien
Djerassiplatz 1
1030 Wien
Austria
Tel.: +43-4277-76513
Email: simon.rittmann@univie.ac.at

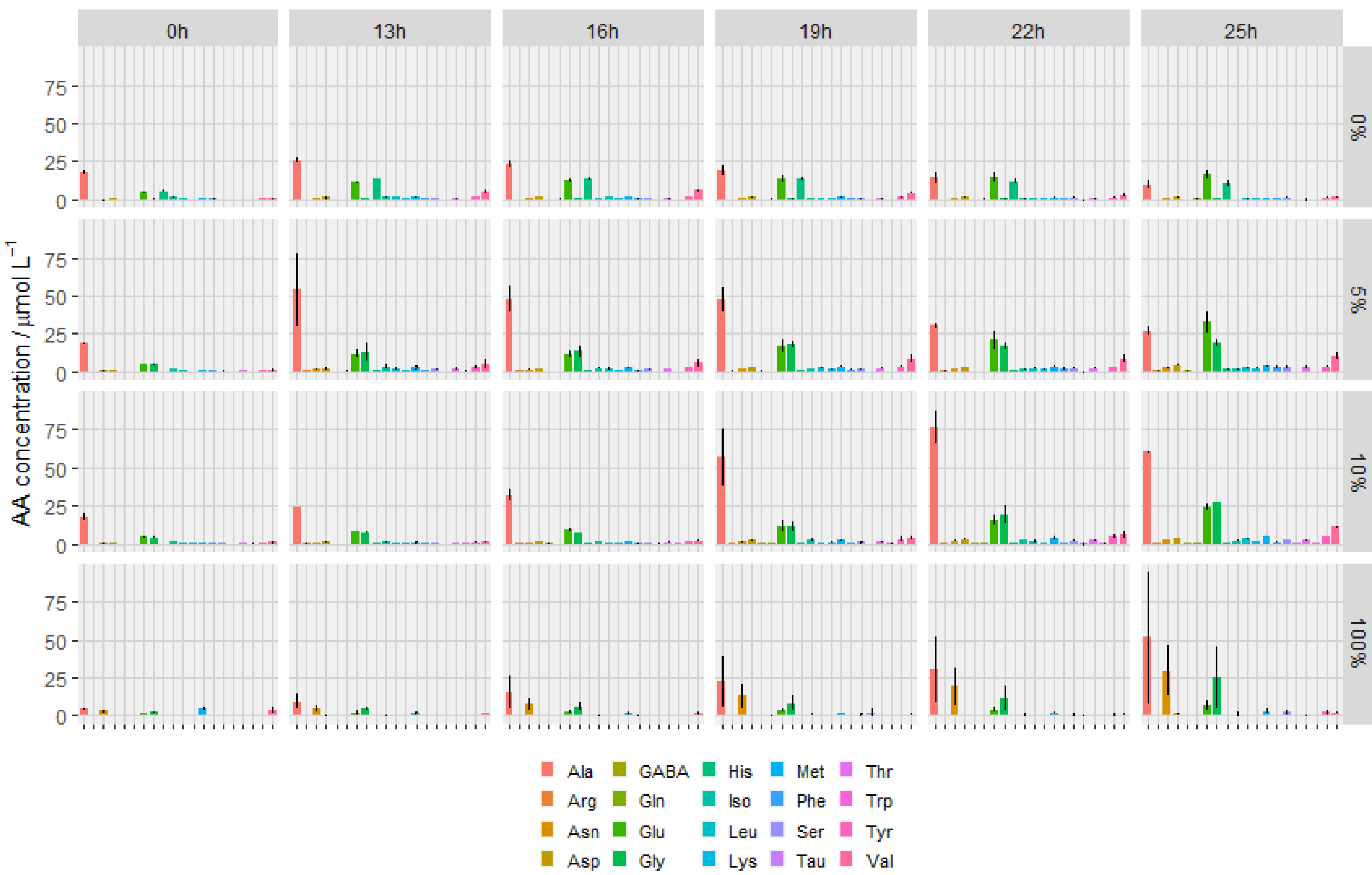

Supplementary figure 1: Proteinogenic amino acid (AA) concentration / µmol L^{-1} of supernatant samples from *M. marburgensis* fed-batch cultivations in media without Carbonate and with $H_2/CO_2/N_2$ (7:1:1). The AA concentrations are shown as individual bar charts with standard deviations for each time point (n = 3). The legend at the bottom of the graph indicates the quantified AAs. On the left-hand y-axis, the AA concentration / µmol L^{-1} is shown. Initial NH_4^+ concentrations of the respective time series are indicated on the right-hand y-axis from top to bottom: 0%, 5%, 10% and 100%. The sampling time is shown on the x-axis as headers from left to right. GABA serves as an internal quantification standard.

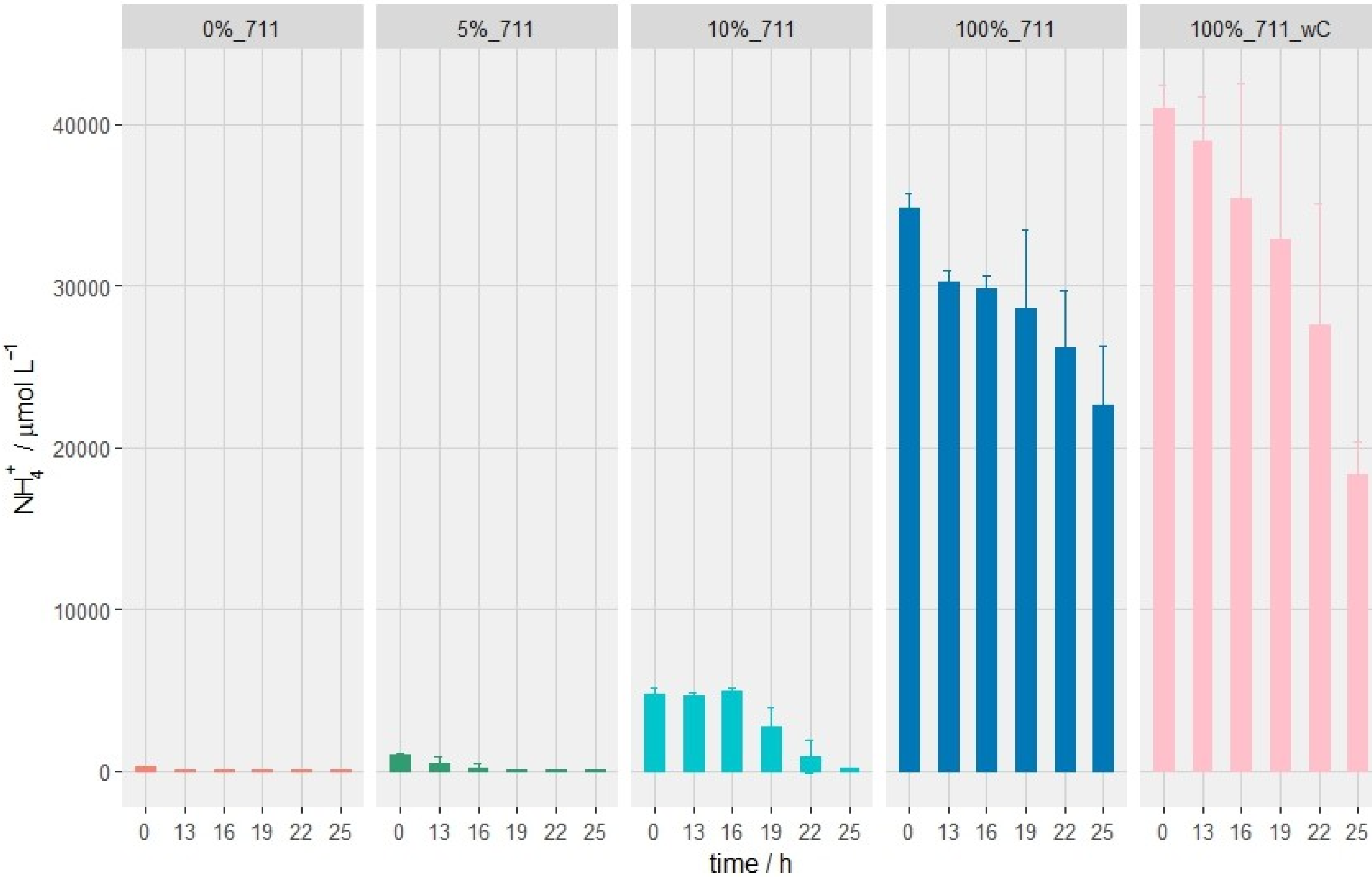

Supplementary figure 2: NH_4^+ concentration (µmol L^{-1}) from fed-batch experiments. Colours indicate the different concentrations of NH_4^+ in the media, 0% (salmon), 5% (green), 10% (turquoise), 100% (blue), and 100% with carbonate (wC) in the media (pink). Time / h is shown on the x-axis for each fed-batch gas fermentation individually. All experiments are n = 3.

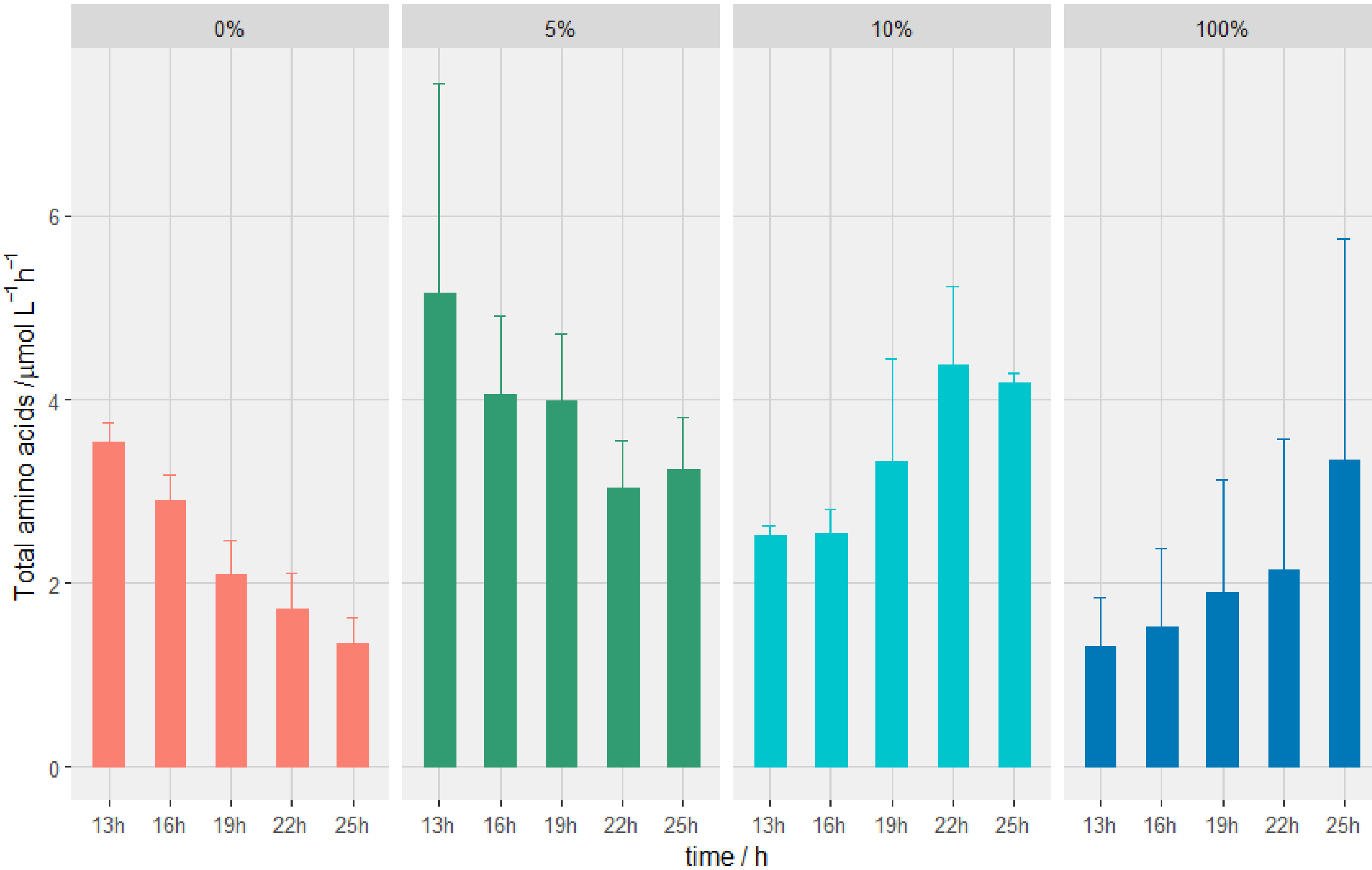

Supplementary figure 3: Total amount of excreted amino acids (total AAs) of fed-batch experiments (n = 3). Colours indicate the different concentrations of NH_4^+ in the media, 0% (salmon), 5% (green), 10% (turquoise) and 100% (blue). Sampling time is indicated on the x-axis.

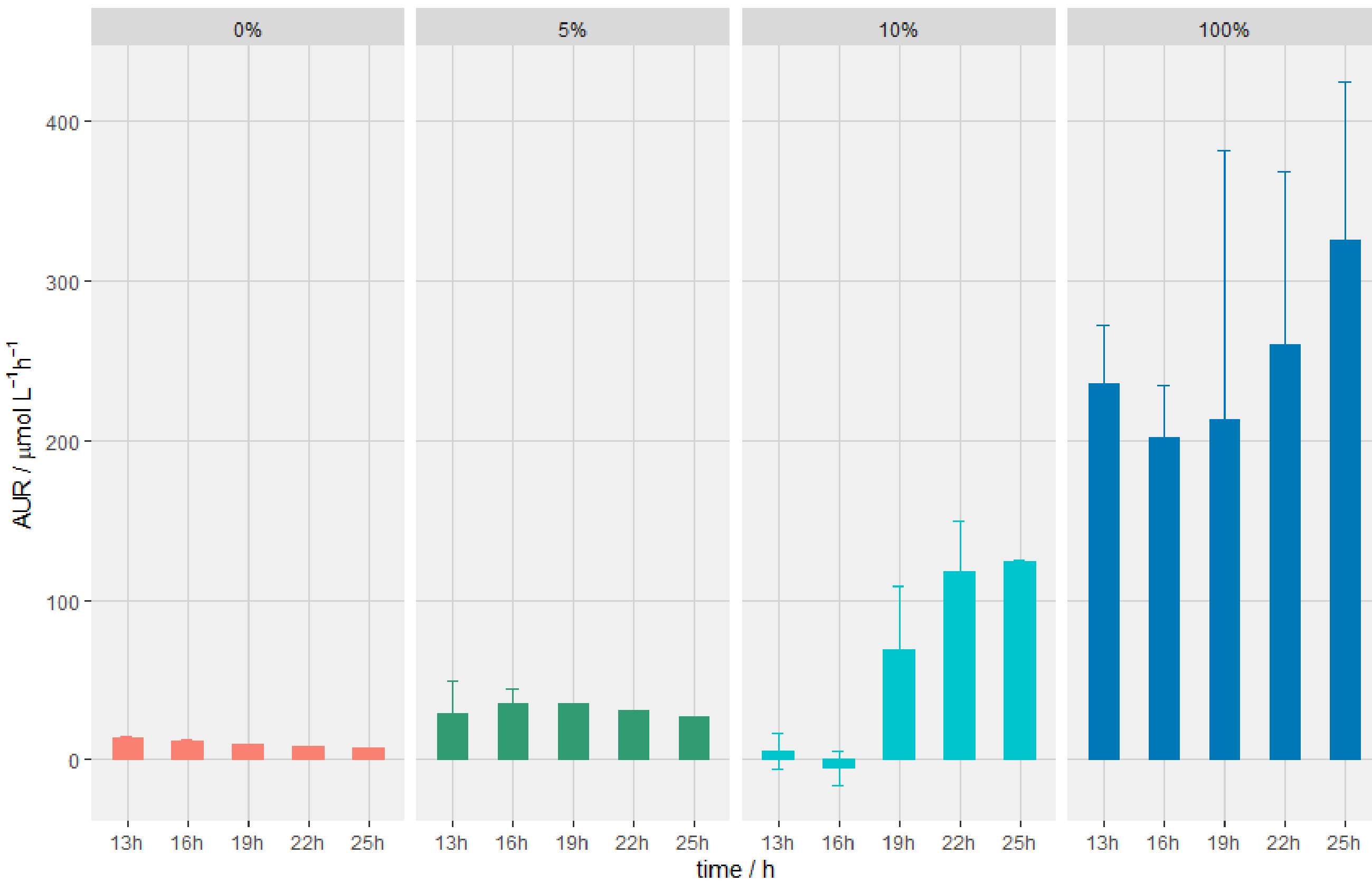

Supplementary figure 4: Ammonium uptake rate (AUR / µmol L^{-1} h^{-1}) of fed-batch experiments with (n = 3). Colours indicate the different concentrations of NH_4^+ in the media, 0% (salmon), 5% (green), 10% (turquoise) and 100% (blue). Sampling time is indicated on the x-axis.

Supplementary table 1: NH_4^+ concentrations

NH_4^+ / %	g L^{-1}	mmol L^{-1}
0	0	0
1	0.02	0.373
5	0.11	2.056
10	0.21	3.926
100	2.10	39.25

Supplementary table 2: Results of Brown-Forsythe and two-way ANOVA for **Ala**

Brown–Forsythe test

Test Statistic = 1.0966

p-value = 0.3725

Two-way ANOVA

	Df	Sum Sq	Mean Sq	F value	Pr(>F)
time	1	5506	5506.5	14.6098	0.0002494 ***
NH_4^+	1	6539	6539.4	17.3504	7.365e-05 ***
time:NH_4^+	1	12	12.3	0.0325	0.8573303
Residuals	86	32414	376.9		

Signif. codes: 0 ‘***’ 0.001 ‘**’ 0.01 ‘*’ 0.05 ‘.’ 0.1 ‘ ’ 1

Supplementary table 3: Results of Brown-Forsythe and two-way ANOVA for **Asn**

Brown–Forsythe test

Test Statistic = 1.3563

p-value = 0.1587

Two-way ANOVA

	Df	Sum Sq	Mean Sq	F value	Pr(>F)
time	1	683.7	683.71	14.668	0.000243 ***
NH_4^+	1	1216.7	1216.71	26.102	1.927e-06 ***
time:NH_4^+	1	517.1	517.14	11.094	0.001277 **
Residuals	86	4008.8	46.61		

Signif. codes: 0 ‘***’ 0.001 ‘**’ 0.01 ‘*’ 0.05 ‘.’ 0.1 ‘ ’ 1

Supplementary table 4: Results of Brown-Forsythe and two-way ANOVA for **Glu**

Brown–Forsythe test

Test Statistic = 1.0067

p-value = 0.4769

Two-way ANOVA

	Df	Sum Sq	Mean Sq	F value	Pr(>F)
time	1	1351.42	1351.42	83.256	2.745e-14 ***
NH_4^+	1	2247.73	2247.73	138.475	< 2.2e-16 ***
time:NH_4^+	1	675.89	675.89	41.639	6.210e-09 ***
Residuals	86	1395.95	16.23		

Signif. codes: 0 ‘***’ 0.001 ‘**’ 0.01 ‘*’ 0.05 ‘.’ 0.1 ‘ ’ 1

Supplementary table 5: Results of Brown-Forsythe and two-way ANOVA for **Gly**

Brown–Forsythe test

Test Statistic = 1.0173

p-value = 0.4639

Two-way ANOVA

	Df	Sum Sq	Mean Sq	F value	Pr(>F)
time	1	1399.2	1399.19	22.1256	9.66e-06 ***
NH_4^+	1	118.9	118.86	1.8795	0.1740
time:NH_4^+	1	19.4	19.39	0.3066	0.5812
Residuals	86	5438.5	63.24		

Signif. codes: 0 '***' 0.001 '**' 0.01 '*' 0.05 '.' 0.1 ' ' 1
